# Dose–response analysis of *Bacillus thuringiensis* HD‐1 cry‐ spore reduction on surfaces using formaldehyde with pre‐germination

**DOI:** 10.1111/jam.15767

**Published:** 2022-10-24

**Authors:** Ehsan Gazi, Marc Bayliss, Christine O'Sullivan, Clare Butler‐Ellis, Brian France, Richard M. Clapperton, Dean Payne, Norman Govan

**Affiliations:** ^1^ Dstl Porton Down Wiltshire UK; ^2^ Silsoe Spray Applications Unit Bedford UK; ^3^ TDA Research Wheat Ridge Colorado USA; ^4^ Battelle UK Ltd Havant, Hampshire UK

**Keywords:** anthrax, *Bacillus anthracis*, biocides, decontamination, dose–response, formaldehyde, germination, spores

## Abstract

**Aim:**

To establish a basis for rapid remediation of large areas contaminated with *Bacillus anthracis* spores.

**Methods and Results:**

Representative surfaces of wood, steel and cement were coated by nebulization with *B. thuringiensis* HD‐1 cry‐ (a simulant for *B. anthracis*) at 5.9 ± 0.2, 6.3 ± 0.2 and 5.8 ± 0.2 log10 CFU per cm^2^, respectively. These were sprayed with formaldehyde, either with or without pre‐germination. Low volume (equivalent to ≤2500 L ha^−1^) applications of formaldehyde at 30 g l^−1^ to steel or cement surfaces resulted in ≥4 or ≤2 log10 CFU per cm^2^ reductions respectively, after 2 h exposure. Pre‐germinating spores (500 mmol l^−1^ l‐alanine and 25 mmol l^−1^ inosine, pH 7) followed by formaldehyde application showed higher levels of spore inactivation than formaldehyde alone with gains of up to 3.4 log10 CFU per cm^2^ for a given dose. No loss in *B. thuringiensis* cry‐ viability was measured after the 2 h germination period, however, a pre‐heat shock log10 reduction was seen for *B. anthracis* strains: LSU149 (1.7 log10), Vollum and LSU465 (both 0.9 log10), LSU442 (0.2 log10), Sterne (0.8 log10) and Ames (0.6 log10).

**Conclusions:**

A methodology was developed to produce representative spore contamination of surfaces along with a laboratory‐based technique to measure the efficacy of decontamination. Dose–response analysis was used to optimize decontamination. Pre‐germinating spores was found to increase effectiveness of decontamination but requires careful consideration of total volume used (germinant and decontaminant) by surface type.

**Significance and Impact of the Study:**

To be practically achievable, decontamination of a wide area contaminated with *B. anthracis* spores must be effective, timely and minimize the amount of materials required. This study uses systematic dose–response methodology to demonstrate that such an approach is feasible.

## INTRODUCTION

*Bacillus anthracis* is the causative agent of the fatal disease anthrax. Intentional release of this bacterium in the 2001 ‘Amerithax’ attacks through the US postal system resulted in five deaths, more than 20 illnesses and a decontamination response that cost hundreds of millions of dollars with facilities that were not reopened for 2 years (Schmitt & Zacchia, 2012); one building was opened after 6 years (American Media Incorporated building at Boca Raton, Florida). However, this was considered a relatively localized release compared with a wide area release, which has greater implications on the environment, economy, essential services, among other areas (Franco & Bouri, 2010).

The attribute that sets *B. anthracis* apart from most other pathogenic microorganisms is its ability to form spores that are highly resistant to external physical and chemical stressors. This ‘hardy’ property is the net result of a combination of factors that include a dehydrated spore core, dipicolinic acid content, acid‐soluble proteins that protect spore DNA and thick layers of cross‐linked coat proteins (Driks, 1999; Riesenman & Nicholson, 2000; Setlow, 2006; Slieman & Nicholson, 2001). These factors impart resistance to environmental stresses such as temperature extremes, desiccation, radiation and decontamination reagents, making them highly persistent in the environment. The ability of *B. anthracis* to persist in the outdoor environment was starkly demonstrated when soil samples taken on Gruinard Island 44 years following release were found to contain viable spores (Manchee et al., 1983). *Bacillus anthracis* has also been isolated from 200 years old animal carcasses found in the northern region of the Kruger National Park in South Africa (De Vos, 1990) and considered indigenous to the KNP ecosystem (Hugh‐Jones & Blackburn, 2009).

To enable Gruinard Island to be returned to public use, vegetation was firstly removed with herbicides and burning. This was followed by extensive mist irrigation with up to 500,000 L ha^−1^ (50 L m^−2^) of formalin (CH_2_O) in seawater (an aqueous solution of formaldehyde) and concentrations of 5%–38% w/w (formaldehyde) to treat 3.7 ha over a 3‐week period (Manchee et al., 1983). While effective, this approach had a high logistical burden and although formaldehyde/formalin was a well‐established broad‐spectrum disinfectant (Centers for Disease Control and Prevention, 2008; Lach, 1990), its modern application in an outdoor environment would raise environmental concerns due to its toxicity and carcinogenic properties.

A microbiological approach that could potentially be exploited to reduce the dose (i.e. quantity per unit area) of decontaminant needed to achieve high levels of spore reduction on surfaces is by pre‐germination of spores (Kohler et al., 2017). Germination is a complex process during which the spore sheds its spore coat and transforms into a vegetative cell (Kohler et al., 2017; Setlow, 2014), when it is more susceptible to environmental stresses (ultraviolet irradiation, temperature, desiccation) and chemically induced inactivation. However, it is unlikely that all spores in a population would be immediately responsive to induction by germinants as some may be ‘superdormant (SD)’ (Kohler et al., 2017; Setlow, 2014). A major reason for superdormancy is thought to be lack of germinant receptors in the inner membrane that are required to trigger a cascade of intracellular and extracellular events that differentiates the spore into its vegetative form (Kohler et al., 2017; Setlow, 2014). Germination is also impacted by spore concentration and temperature (Buhr et al., 2019; Titball & Manchee, 1987).

For *B. anthracis*, nutrient germinants are primarily amino acids and purine nucleosides, where inosine or l‐alanine typically serve as primary germinants (Fisher & Hanna, 2005). l‐alanine can trigger germination of spores by itself, although only at concentrations above 30 mmol l^−1^ (Fisher & Hanna, 2005; Kohler et al., 2017). Inosine can be synergistically paired with a variety of l‐amino acids as well as with the primary germinant l‐alanine (Fisher & Hanna, 2005).

The increased susceptibility of *B. anthracis* to a range of oxidative decontaminants and formaldehyde following germinant induction was demonstrated in suspension tests (Celebi et al., 2016; Omotade et al., 2014). Previous researchers inoculated impermeable glass surfaces with *B. thuringiensis* or *B. anthracis* spore suspensions by pipette and then exposed them to germinant (10 mmol l^−1^ l‐alanine and 10 mmol l^−1^ inosine) followed by 1% hydrogen peroxide (Omotade et al., 2014). Pre‐germination resulted in a greater level of spore inactivation compared to using the decontaminant alone under idealized conditions (Omotade et al., 2014).

The present study builds on this previous work (Celebi et al., 2016; Omotade et al., 2014) by evaluating the effect of pre‐germination on the decontaminant (formaldehyde) dose (kg ha^−1^) needed to achieve spore inactivation on different surfaces inoculated with *B. thuringiensis* subsp. kurstaki HD‐1 cry‐. Dose–response methodology is critical in establishing practical formulation regimes for wide area application as it allows the total volume of liquid to be minimized while maintaining efficacy.

We used *B. thuringiensis HD‐1 cry‐* as a simulant for *B. anthracis* as it is regarded as the same species at the genomic level (Daffonchio et al., 2000; Helgason et al., 2000) while also having highly comparable spores in terms of size and integuments (composition and morphology) (Tufts et al., 2014). Both *B. anthracis* and *B. thuringiensis* have an exosporium, which is a hydrophobic surface that is separated from the spore coat and one or more paracrystalline layers surrounded by an outermost ‘hairy nap’ layer (Ball et al., 2008). *Bacillus thuringiensis* is thus preferred over other surrogate spores, such as *B. atrophaeus* and *subtilis*, which lack the exosporium (Carrera et al., 2006). *B. atrophaeus* and *subtilis* are the same organism and also commonly referred to by their historical designation of *B. globigii* or simply BG (Burke et al., 2004).

This Hazard group 1, non‐insecticidal cry‐ strain was developed for use in outdoor trials (Bishop & Robinson, 2014), which supported our aim to develop a basis for the rapid remediation of large outdoor areas contaminated *B. anthracis*. To this end, we use ha (10,000 m^2^) as a unit of measurement for formulation surface coverage throughout this manuscript.

Finally, we investigated if the pre‐germination strategy using our formulation and simulant strain could be extrapolated with confidence to virulent strains of *B. anthracis* (containing the pX01 and pX02 virulence plasmids) and the vaccine strain Sterne (containing pX01 only).

## METHODS

### Preparation of bacterial strains

A loop of the respective *Bacillus* culture stored frozen at −70°C (*B. thuringiensis* cry‐ and the seven *B. anthracis* strains; Sterne; LSU149; LSU463; Ames, Vollum; LSU465 and LSU 442) was streaked onto L‐agar plates and incubated overnight at 37°C to isolate single colonies. Three single colonies from each *Bacillus* were then picked and suspended into 100 ml of pre‐warmed L‐broth in 500‐ml conical flasks. The flasks were placed on an orbital shaker set at a temperature of 37°C and shaken at 180 rpm for 6 h after which 5 ml of each culture was inoculated into 24 roux flasks containing 75 ml Nutrient Sporulation Media (NSM) and incubated at 37°C for 10 days. Sporulation was confirmed by taking a small size and observing the presence of phase bright spores by phase contrast microscopy. Spores were harvested from each roux flask into 15 ml of sterile distilled water (SDW) and centrifuged at 10,000 *g* for 15 min. After discarding the supernatant the pellet was re‐suspended in SDW by vortexing to wash the spores. This wash step of centrifugation and re‐suspension was performed a further three times. The final washed pellets from each *Bacillus* were re‐suspended and combined into approximately 100 ml of SDW resulting in a concentration of approximately 1 × 10^9^ CFU per ml. The Hazard Group 3 *Bacillus anthracis* strains were handled by trained and experienced Dstl staff in specialist facilities at Containment Level 3.

### Preparation of formulations

#### Germinant

A final concentration of 500 mmol l^−1^ l‐alanine (CAS 56–41‐7) and 25 mmol l^−1^ inosine (CAS 58‐63‐9) was prepared in SDW and adjusted to pH 7 using 67 mmol l^−1^ sodium phosphate dibasic dihydrate. A surfactant blend of 1:1 Synperonic PE L62 and PE L64 (0.2% w/w) was added only for surface testing. All chemicals were purchased from Sigma‐Aldrich, except for SDW (VWR), Synperonic PE L62 and PE L64 (Croda).

#### Decontaminant

A 37% w/w formaldehyde solution (formaldehyde) (Sigma Aldrich) was diluted in SDW to make stock solutions with final concentrations of 1%, 3%, 15%, 20% and 30% w/w (equivalent to 10, 30, 150, 200 and 300 g l^−1^). Each stock solution contained 0.2% w/w 1:1 Synperonic PE L62/L64.

### Test surfaces

Steel (grade 316, 20 [l] × 20 [w] × 1.5 [d] mm), pinewood (22 [l] × 18 [w] × 2 [d] mm) and cement (circular discs of 21 mm × 5 mm [h]) were sterilized by autoclave at 121°C for 30 min. Cement was produced in‐house by the following procedure: Water was mixed with cement powder (Portland—limestone cement [Standard: CEM 11A‐LL 32.5 R]) at a 0.35 weight ratio of water:cement powder for 5 min using a combination of mechanical stirring (Heidolph R2R 2050 Electronic operating at 200 rpm) and hand stirring using a spatula. This produced a paste that could be poured into a polycarbonate mould (the bottom covered with parafilm). The mould was picked up and lightly tapped against the preparation base so that air from the bulk was expelled. The process resulted in water rising to the surface of the cement, which then evaporated/absorbed back into the cement column as it dried over several days. The cement column was released from the polycarbonate mould following a drying period of 4 days at 20°C. The cement columns were then sliced into 5 mm (height) discs using a tile cutter. The sterile test surfaces were inoculated by nebulization (see Results and Discussion).

### Suspension tests

In triplicate, 9‐part germinant concentrate was added to 1‐part spores to give a final concentration of 10^7^ CFU per ml of each *Bacillus* strain and final germinant concentrations of l‐alanine (500 mmol l^−1^) and inosine (25 mmol l^−1^) at pH 7. Spores were incubated for 2 h at 21°C before removing a 100 μl aliquot to produce a dilution series in SDW for immediate plating onto tryptic soy agar (TSA) and incubation at 31°C to determine total viable counts. Samples at appropriate dilutions were also heat‐shocked at 65°C for 30 min before plating and spore enumeration. Culture plates with counts of between 10 and 100 CFU were recorded. The number of germinated spores was determined by calculating the difference between heat‐shocked and non‐heat‐shocked plate counts. Control samples consisted of spores incubated in distilled water.

### Surface tests

**FIGURE 1:**
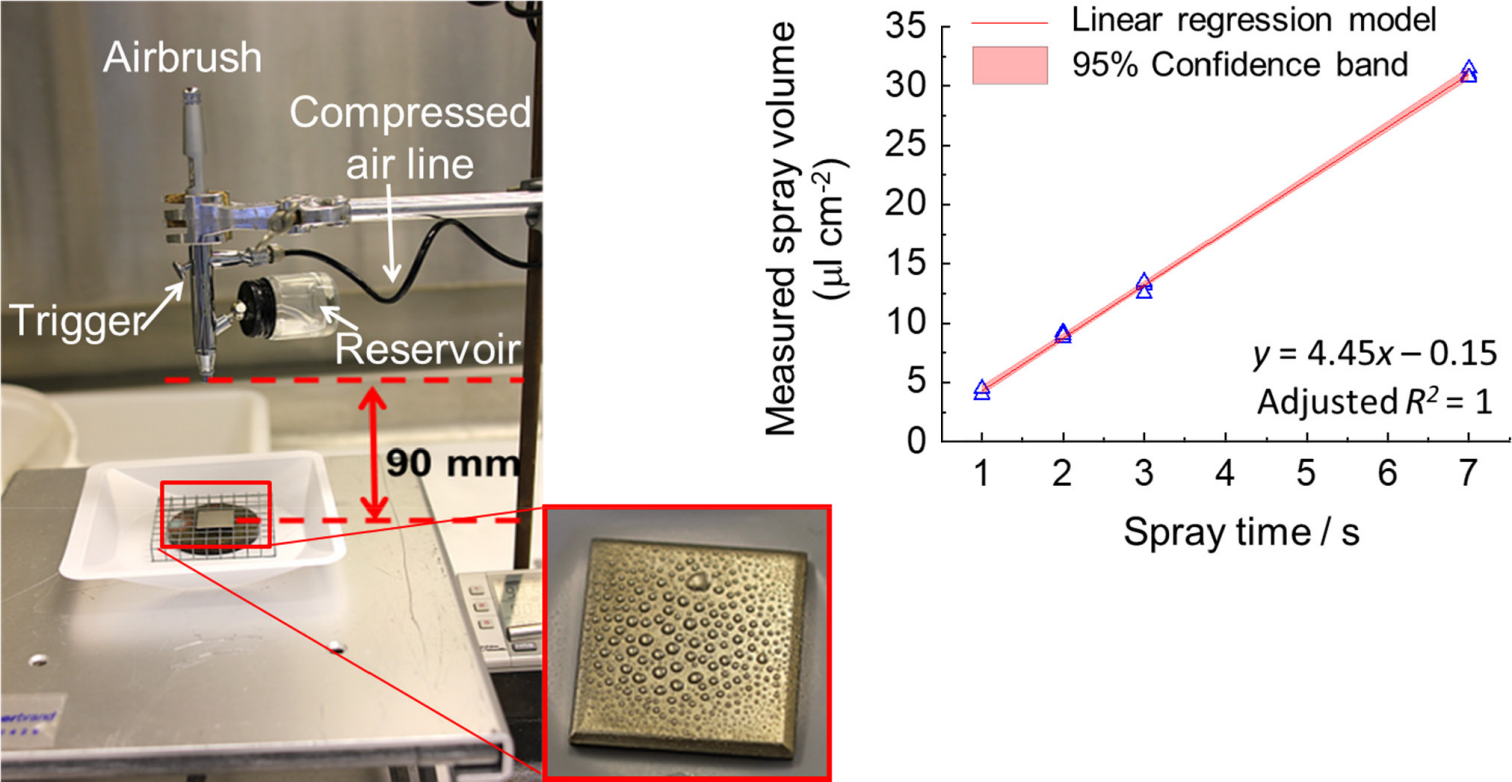
Photographs of the experimental setup for the manually triggered laboratory‐scale spray applicator and a steel surface sprayed with water to show droplet distribution. The graph shows a linear regression model with ±95% CI bands plotted against increasing spray volume and time when applied to a wood surface.

Treatment with decontaminant by spray application represented the most likely method of delivery if a large area was contaminated. A laboratory‐scale spray applicator (LSSA) system was developed (Figure 1) based on a commercial airbrush system (SprayCraft SP50K). The airbrush was connected to a laboratory compressed airline via a flow meter and vinyl hose. The flow meter was needed to measure and regulate airflow. A glass bottle (liquid reservoir) was filled with decontaminant solution and secured to the airbrush. The spray was dispensed by firstly pressing down on the trigger to enable airflow (3.5 L min^−1^) and then pulling back on the trigger to dispense the formulation (the reduction in air pressure in the sampling pipe draws the fluid through the nozzle by the Venturi effect). The airbrush was securely clamped to a retort stand and orientated to the target surface inoculated with *B. thuringiensis* cry‐ spores. As the force of the airflow could push deposited fluid away from the centre of the steel surface towards the edges the distance between the airbrush nozzle and the test surface, as well as the airflow rate, were optimized to reduce this effect and ensure that the sprayed liquid deposited onto the surface uniformly. A control test demonstrated the viability of *B. thuringiensis* cry‐ spores was not significantly affected by suspension in SDW for 24 h compared with 0 h sample (*p* = 0.09 at 95% CI; mean spore concentration at 0 h was 7.3 ± 0.03 CFU per ml, at 24 h it was 7.4 ± 0.03 CFU per ml). An additional control test was performed to ensure spores on the surface were not mobilized by the force of the airflow itself, by using a continuous ‘dry’ spray time of 8 s at 5 L min^−1^ (i.e. greater than the flow used in experiments). There was no statistical difference at the 95% CI in the number of spores recovered from the steel or wood coupons following this treatment compared with the population control (i.e. untreated nebulized coupons); *p* = 0.25 for steel coupons and *p* = 0.39 for wood coupons.

The spray delivery onto coupons was calibrated by measuring the quantity on each coupon gravimetrically for different spray durations. This demonstrated that reproducible spray volumes of 4–70 μl cm^−2^ (equivalent to 400–7000 L ha^−2^) could be delivered. Pre‐weighed inoculated surfaces were sprayed with the decontaminant for the required spray time before being reweighed to calculate the actual volume deposited (μl cm^−2^). Doses could be manipulated by changing either the spray duration or the concentration, or both. The test surfaces were transferred to individual wells of a six‐well plate, lidded and then incubated at 21°C during a defined exposure. Following this period, test surfaces were transferred into 50 ml Falcon tubes containing 8 ml Dey Engley broth (Sigma Aldrich) to neutralize residual formaldehyde. One gram of sterile sand was added to the Falcon tube and vortexed for 30 s to mobilize spores from the surface and into the DEB media. After the sand settled, a dilution series in PBS was prepared for plating onto TSA and incubation at 31°C to determine total viable counts as described above.

For experiments that involved germination prior to decontamination, the inoculated surfaces were immersed in 4 ml of germinant solution so that it was saturated. This ensured that maximum germination was achieved on a surface during the 2 h incubation period. Excess germinant was drained and blotted‐off by touching the edge of the surface to filter paper before the surface was sprayed using the LSSA with formaldehyde. Spore recovery was performed as described above.

### Statistics

All statistical analyses were performed using Minitab® 19.1. Data normality was assessed using probability QQ‐plots at 95% CI, prior to conducting one‐way analysis‐of‐variance (ANOVA) using the *F*‐statistic to test for significant differences between the mean measurements of two or more groups, followed by a post‐hoc Tukey test for significant differences between the means of subsets of groups. Note that one‐way ANOVA was performed following a non‐significant Levene's test to ensure data homogeneity (*p* ≤ 0.05). Prior to performing independent two‐sample *T*‐test's, equality of variance were assessed using the Bartlett's test (if data were drawn from normal distribution) or Levene's test (if data were drawn from skewed distribution), where a value of *p* ≤ 0.05 indicated equal variance between independent variables. All ±error measurements are stated as standard deviation.

## RESULTS

### Spore nebulization onto surfaces

Surfaces were inoculated with *B. thuringiensis* cry‐ spores by aerosol deposition using a medical nebulizer (Omcrom NE‐C28‐E) to prepare a realistic surface contamination. The approach was verified in a two‐step approach: Step 1 used an aqueous dye (green ‘S’ dissolved in water) to calculate and optimize a theoretical spore density based on the volume of aerosolised droplets deposited across the inoculation area; Step 2 verified this theoretical deposition using *B. thuringiensis* cry‐ spores.

**FIGURE 2:**
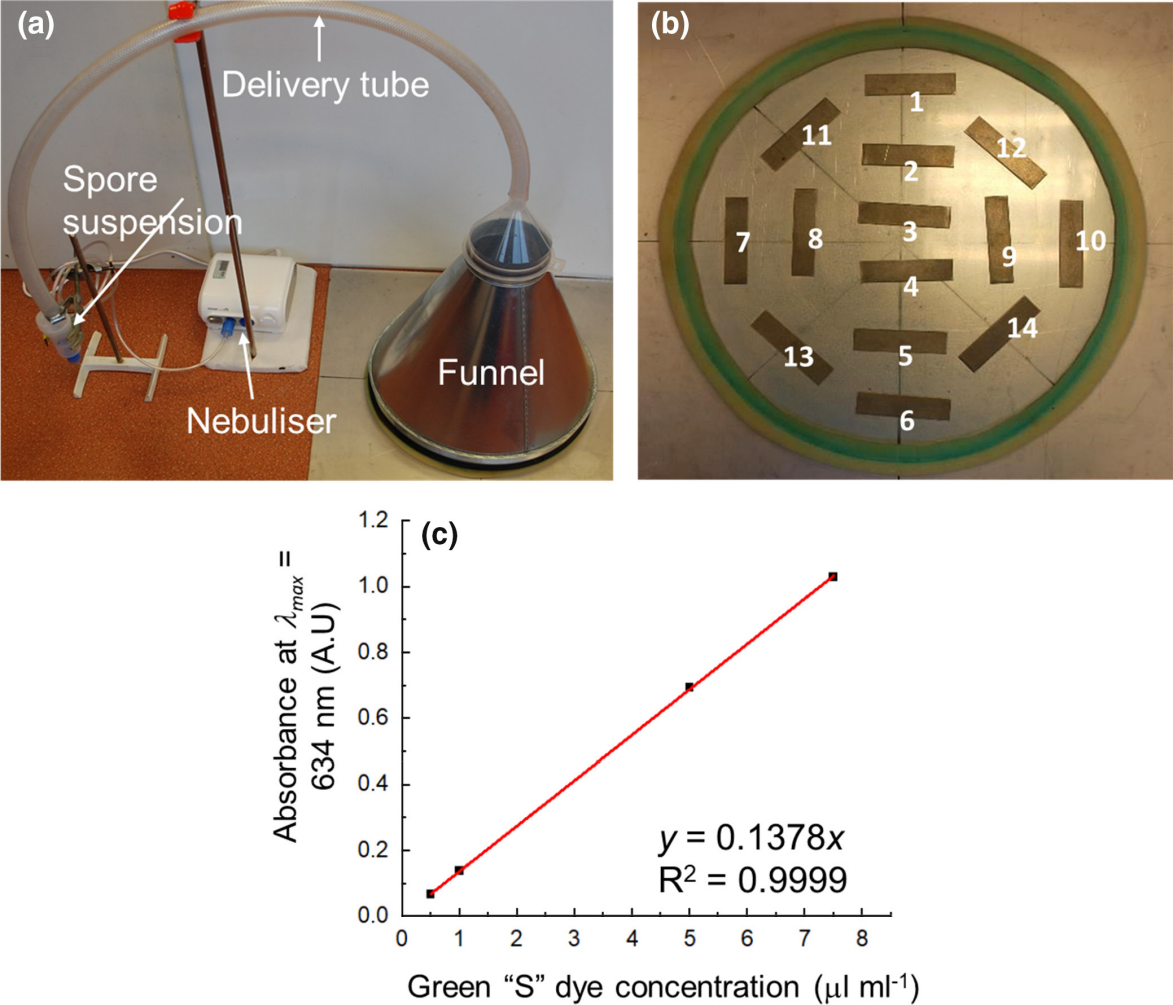
(a) Photograph showing configuration of the nebulizer system to generate and deposit aerosolised *Bacillus thuringiensis* cry‐ spores or dye onto surfaces; (b) layout of stainless steel surfaces (each 16 cm^2^) within the deposition field under the funnel canopy for dye droplet deposition measurements; (c) UV–visible calibration curve relating absorbance at 634 nm with Green ‘S’ concentration (μl ml^−1^).

The nebulizer produced an aerosol of dye droplets from a 5 ml dye solution run for 20 min. The droplets were carried via a delivery tube to a funnel, where 16 cm^2^ steel surfaces (*n* = 14) were laid across the deposition field (Figure 2a,b). The volume of dye solution deposited as a function of surface position was calculated by washing dye off the surface with 10 ml distilled water and then taking an absorbance measurement at *λ*_max_ = 634 nm (Table 1). These measurements were related to deposited volumes using the calibration graph shown in Figure 2c.

**TABLE 1:** Volume of dye solution deposited as a function of surface position within the target area

Position	Wash‐off volume (ml)	Absorbance at *λ*_max_ = 634 nm (A.U)	Green ‘S’ concentration (μl ml^−1^)	Recovered dye volume (μl), corrected for dilution factor
1	10	0.236	1.71	17.13
2	10	0.241	1.75	17.51
3	10	0.243	1.76	17.60
4	10	0.237	1.72	17.23
5	10	0.244	1.77	17.74
6	10	0.239	1.73	17.31
7	10	0.232	1.68	16.81
8	10	0.243	1.76	17.63
9	10	0.246	1.78	17.84
10	10	0.239	1.73	17.31
11	10	0.236	1.71	17.13
12	10	0.235	1.70	17.04
13	10	0.243	1.77	17.66
14	10	0.235	1.71	17.06

**FIGURE 3:**
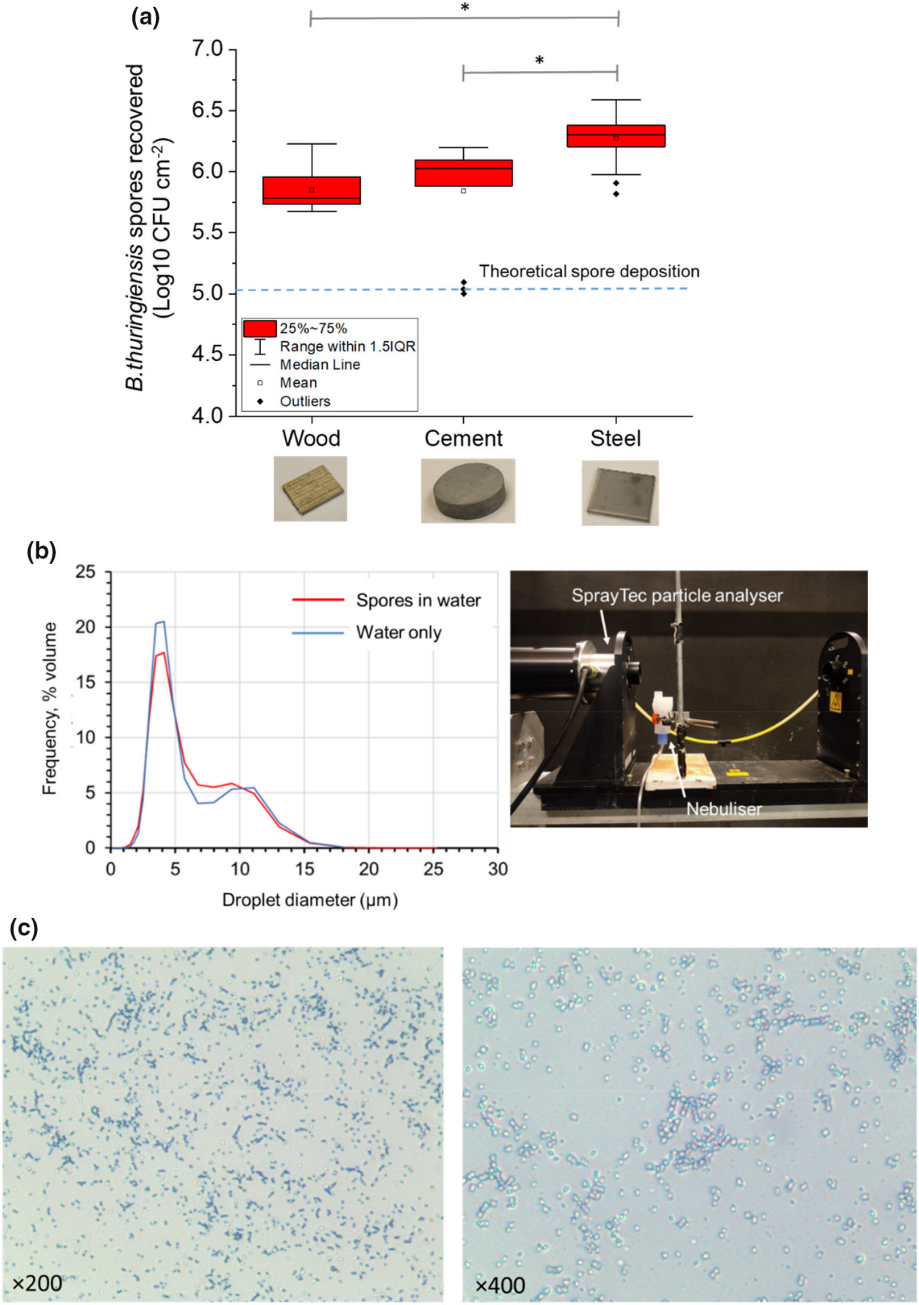
(a) Recovery of *Bacillus thuringiensis cry‐* spores from steel (*n* = 21), cement (*n* = 15) and wood (*n* = 21) surfaces following nebulization (where * indicates *p* ≤ 0.05 using one‐way ANOVA with multiple comparisons). The dotted‐line denotes the theoretical spore deposition calculated from nebulized dye (5.03‐log10 CFU per cm^2^). (b) Frequency of droplet size distribution by %volume, from nebulizing water or *B. thuringiensis cry‐* spores in water (2.8 × 10^10^ CFU per ml) using the Omcrom NE‐C28‐E system. The experimental set‐up for these droplet measurements is shown in the photograph: A SprayTec laser droplet analyser was used, where the distance between the nozzle tip and the center of the lazer beam was 45 mm and the sampling time was 90 s. (c) Representative photomicrographs showing spore inoculum (10^8^ CFU per ml) nebulized onto glass taken at ×200 and ×400 magnification.

A one‐sample *T*‐test confirmed that the volume deposited between each surface was not significantly different to the deposited mean of 17.36 μl (*p* = 1.00 at 95% CI), where the mean volume deposited per unit area was 1.09 ± 0.02 μl cm^−2^. This dye volume per unit area on steel (1.09 ± 0.02 μl cm^−2^) was used to calculate the theoretical number of spores deposited, based on the initial inoculum nebulized (1 × 10^8^ CFU per ml), and then compared with experimental data on spore recovery (Figure 3).

Operating the nebulizer in the same way as in the dye experiments, spores were deposited onto surfaces of steel, wood and cement by nebulizing 5 ml of an aqueous spore suspension with a concentration of 1 × 10^8^ CFU per ml for 20 min. Figure 3a shows a box‐chart comparing spore recoveries between each surface and the calculated theoretical spore deposition based on the dye experiment.

The theoretical value obtained by the dye measurement was subsequently verified against empirical measurements of spore recoveries from nebulized steel, wood and cement surfaces (Figure 3) using the developed nebulization parameters. It was found that all surfaces resulted in higher recoveries (+0.9 to 1.2 log10 CFU per cm^2^) compared with the theoretical spore deposition (5.03 log10 CFU per cm^2^, dotted line in Figure 3a).

Figure 3b shows the droplet size distribution by %volume, from nebulizing water or *B. thuringiensis cry‐* spores in water (2.8 × 10^10^ CFU per ml) using the Omcrom NE‐C28‐E system. The data were collected by laser diffraction with a SprayTec droplet analyser (Malvern Panalytica) and using the equipment configuration shown in the photograph of Figure 3b.

The nebulized droplet size distribution was comparable with or without spores. Table 2 shows the 10th, 50th and 90th percentile of the cumulative volume distribution, respectively, where the median volume diameter when nebulizing spores was 4.26 μm and water only control was 4.15 μm. Given the average size of single *B. thuringiensis* cry‐ spores was reported by Buhr et al. (2016) to be 1.2 μm, the majority of droplets were larger (98% of droplet sizes had a diameter ≥2 μm). These droplets could therefore hold larger spore aggregates; direct analysis of the particle size distribution of spores in the suspension nebulized was not performed. However, Figure 3c shows representative photomicrographs of spore inoculum (10^8^ CFU per ml) nebulized onto glass taken at ×200 and ×400 magnification. The images show largely dispersed spores as a single layer with some areas of aggregation.

**TABLE 2:** Droplet size distribution data from nebulizing water or spores (2.8 × 10^10^ CFU per ml) using the Omcrom NE‐C28‐E system. Dv10.0, 50.0 and 90.0 corresponds to the 10th, 50th and 90th percentile of the cumulative volume distribution, respectively

	Dv (10.0) (μm)	Dv (50.0) (μm)	Dv (90.0) (μm)	%Volume in different size categories (μm)									
				<02	02‐04	04‐06	06‐08	08‐10	10‐12	12‐14	14–16	16–18	>18
Bt spores	2.77	4.26	9.48	2.02	41.6	30.4	10.2	7.8	5.5	1.8	0.5	0.2	0
Water	2.86	4.15	9.66	0.97	44.9	31.2	7.5	6.7	6	2.1	0.5	0.1	0

We observed a small but significantly lower mean recovery of −0.4 log10 CFU per cm^2^ (at the 95% CI) from porous wood (mean 5.9 ± 0.2 log10 CFU per cm^2^) or cement (mean 5.8 ± 0.2 log10 CFU per cm^2^) surfaces compared with non‐porous steel (mean 6.3 ± 0.2 log10 CFU per cm^2^).

### Sporicidal efficacy of formaldehyde on porous and non‐porous surfaces

**FIGURE 4:**
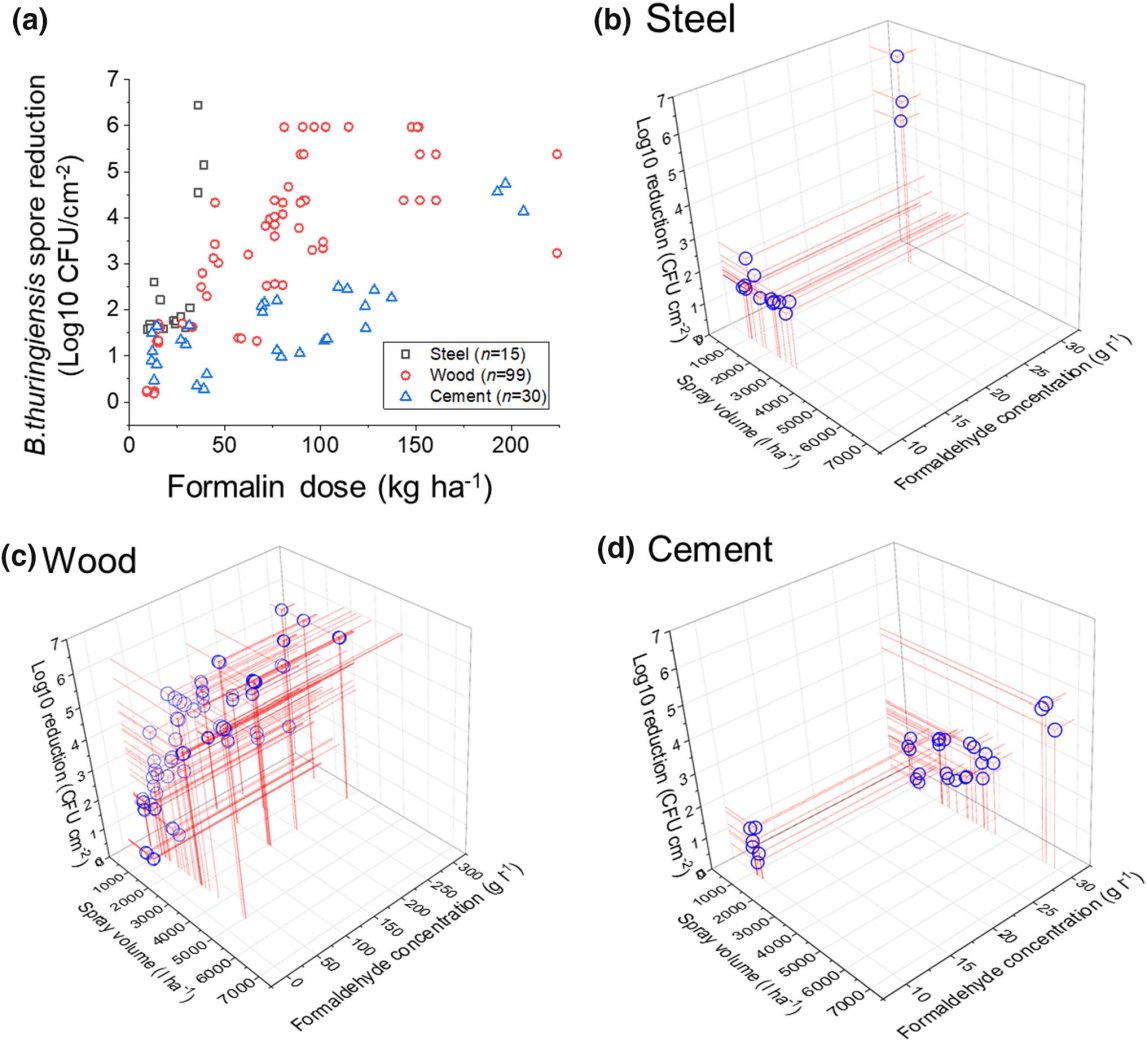
(a) *Bacillus thuringiensis cry‐* spore log10 reduction measured on steel (initial loading: 6.3 ± 0.2 log10 CFU per cm^2^), wood (initial loading: 5.9 ± 0.2 log10 CFU per cm^2^) and cement surfaces (5.8 ± 0.2 log10 CFU per cm^2^) following 2 h treatment to increasing formaldehyde doses (kg ha^−1^) applied using the LSSA; 3D scatter plots showing the relationship between formaldehyde concentration (g l^−1^), application volume (l ha^−1^) and spore killing (log10 reduction) on steel (b), wood (c) and cement (d) surfaces.

Figure 4a shows viable spore reduction measured on steel, wood and cement surfaces following 2 h treatment with formaldehyde doses, ranging from 9 to 921 kg ha^−1^, using the LSSA. The formaldehyde doses ([spray volume (l ha^−1^) × formaldehyde concentration (g l^−1^)]/1000) delivered to each surface were derived through a wide combination of spray volumes (500–6880 L ha^−1^) and formaldehyde concentrations (10–300 g l^−1^). Figure 4b–d show three‐dimensional scatterplots of the log10 reductions (CFU per cm^2^) achieved on each surface type for a given formaldehyde dose. Table 3 shows a summary of the formalin dose–response data for each surface calculated from the raw data points used to produce Figure 4b–d and shows the number of repeat measurements used to measure log10 reduction for a given concentration and target volume pairing.

**TABLE 3:** Mean *Bacillus thuringiensis cry‐* spore log10 reduction measured on steel (initial loading: 6.3 ± 0.2 log10 CFU per cm^2^), wood (initial loading: 5.9 ± 0.2 log10 CFU per cm^2^) and cement surfaces (5.8 ± 0.2 log10 CFU per cm^2^) following 2 h treatment to increasing formaldehyde doses (kg ha^−1^) applied using the laboratory‐scale spray applicator. These data are calculated from the raw data points used to produce Figures 4b–d

Surface	Concentration (g l^−1^)	Mean target volume (L ha^−1^)	Mean formalin dose (kg ha^−1^)	Mean log10 reduction (CFU per cm^2^)	*n* sample number
Steel	10	1042 ± 80	10 ± 1	1.6 ± 0.1	3
	10	1567 ± 243	16 ± 2	2.1 ± 0.5	3
	10	2392 ± 63	24 ± 1	1.8 ± 0.1	3
	10	2942 ± 238	29 ± 2	1.8 ± 0.2	3
	30	1233 ± 58	37 ± 2	5.3 ± 1.0	3
Wood	10	925 ± 43	9 ± 0	0.2 ± 0.0	3
	10	1292 ± 29	13 ± 0	0.2 ± 0.1	3
	30	507 ± 18	15 ± 1	1.5 ± 0.2	6
	30	1125 ± 143	34 ± 4	2.0 ± 0.6	5
	30	1474 ± 67	44 ± 2	3.4 ± 0.8	6
	30	2038 ± 145	61 ± 4	1.8 ± 0.9	4
	30	2600 ± 176	78 ± 5	4.9 ± 1.0	5
	30	3016 ± 40	90 ± 1	4.8 ± 0.8	7
	30	3491 ± 306	105 ± 9	6.0 ± 0.0	3
	30	5000 ± 66	150 ± 2	6.0 ± 0.0	3
	150	513 ± 19	77 ± 3	3.5 ± 0.8	9
	150	1512 ± 28	227 ± 4	4.6 ± 1.2	9
	150	3055 ± 58	458 ± 9	5.0 ± 0.8	9
	200	498 ± 16	100 ± 3	3.4 ± 0.1	3
	200	1502 ± 16	305 ± 3	4.0 ± 0.1	3
	200	3052 ± 59	610 ± 12	4.8 ± 1.1	3
	300	512 ± 21	154 ± 6	4.9 ± 0.5	6
	300	1502 ± 14	450 ± 4	5.4 ± 0.0	6
	300	3033 ± 23	910 ± 7	5.4 ± 0.0	6
Cement	10	1175 ± 25	12 ± 0	1.2 ± 0.3	3
	10	1389 ± 87	14 ± 1	1.0 ± 0.6	3
	30	180 ± 34	5 ± 1	1.0 ± 0.6	6
	30	2464 ± 152	74 ± 5	1.8 ± 0.6	6
	30	3455 ± 313	104 ± 10	1.7 ± 0.7	5
	30	4273 ± 215	128 ± 6	2.1 ± 0.4	4
	30	6623 ± 233	199 ± 7	4.5 ± 0.3	3

Figure 4a with Table 3 show that a ≥4‐log10 reduction in viable *B. thuringiensis cry‐* spores was achieved on non‐porous steel surfaces using mean doses ≥37 ± 2 kg ha^−1^. However, on the wood and cement surfaces, ≥4‐log10 reduction was only measured with relatively higher mean doses of ≥78 ± 5 and ≥199 ± 7 kg ha^−1^, respectively.

### Sporicidal efficacy of formaldehyde with pre‐germination on non‐porous surfaces

**FIGURE 5:**
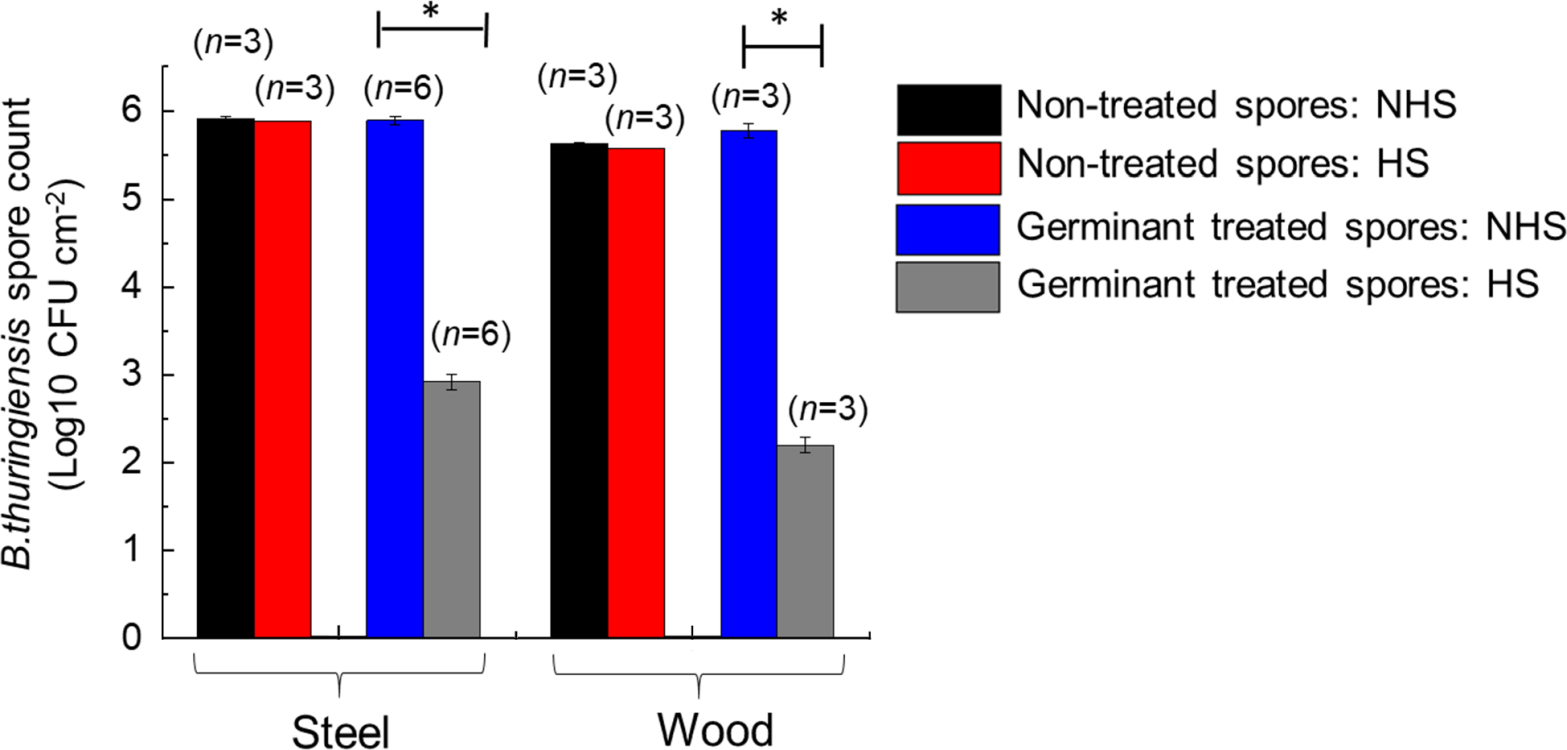
Heat‐shock or non‐heat‐shock counts of *Bacillus thruingiensis cry‐* spores recovered from steel and wood surfaces, with or without, germinant treatment for 2 h (*p ≤ 0.05 one‐way ANOVA with multiple comparisons).

Figure 5 shows that spores treated by immersing inoculated steel or wood surfaces in germinant formulation (500 mmol l^−1^ l‐alanine and 25 mmol l^−1^ inosine at pH 7) for 2 h resulted in a significant fall in recoveries of viable spores following heat‐shock on both steel and wood surfaces (*p* ≤ 0.05). Mean germination rates of 2.8 ± 0.03 and 3.3 ± 0.77 log10 CFU per cm^2^ were measured on steel and wood, respectively. Recoveries from untreated steel surfaces followed by either heat‐shock treatment or no heat‐shock demonstrated mean counts of 5.91 ± 0.03 and 5.88 ± 0.02 log10 CFU per cm^2^, respectively. Recoveries from untreated wood surfaces followed by either heat‐shock treatment or no heat‐shock demonstrated mean counts of 5.62 ± 0.01 and 5.57 ± 0.02 log10 CFU per cm^2^, respectively.

**FIGURE 6:**
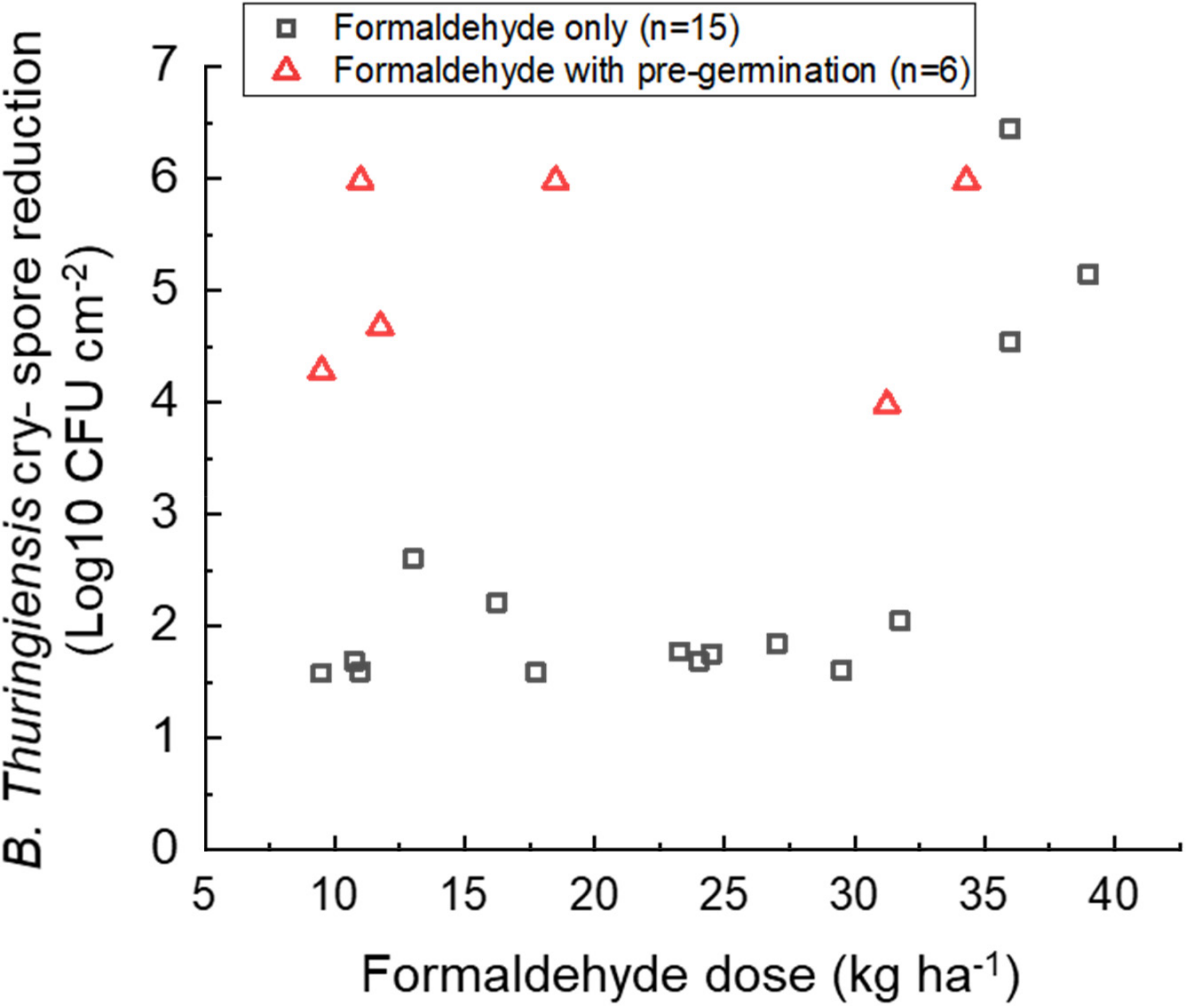
Comparison of *Bacillus thuringiensis cry‐* spore log10 reductions on steel with and without pre‐germination.

Figure 6 shows the loss in spore viability after treatment with germinant for 2 h by immersion and then sprayed with different formaldehyde doses on steel. Note that a subset of the data shown in Figure 4a was reproduced in Figure 6 so that a direct comparison with formaldehyde doses without pre‐germination can be more readily visualized. Table 4 details the formaldehyde concentration and applied volumes used to deliver formaldehyde doses to *B. thuringiensis cry‐* inoculated steel surface following the 2 h pre‐germination step. The data collected from steel surfaces (Figure 6) showed a general trend of greater loss in log10 viability (5.0 ± 0.9 rather than 1.8 ± 0.3 CFU per cm^2^) with germinated compared with ungerminated spores, for formaldehyde doses of <31 kg ha^−1^ (mean difference 3.4 log10, *ρ* = 0.00). Although limited data were collected at higher formaldehyde doses (≥31 kg ha^−1^), there was indication that the gains obtained from germination were reduced at these doses.

**TABLE 4:** Formaldehyde concentration and applied volume used to deliver formaldehyde doses to *Bacillus thuringiensis cry‐* inoculated steel surface shown in Figure 6

Concentration (g l^−1^)	Volume (L ha^−1^)	Formalin dose (kg ha^−1^)
10	950	10
10	1100	11
10	1175	12
10	1850	19
10	3125	31
10	3430	34

**FIGURE 7:**
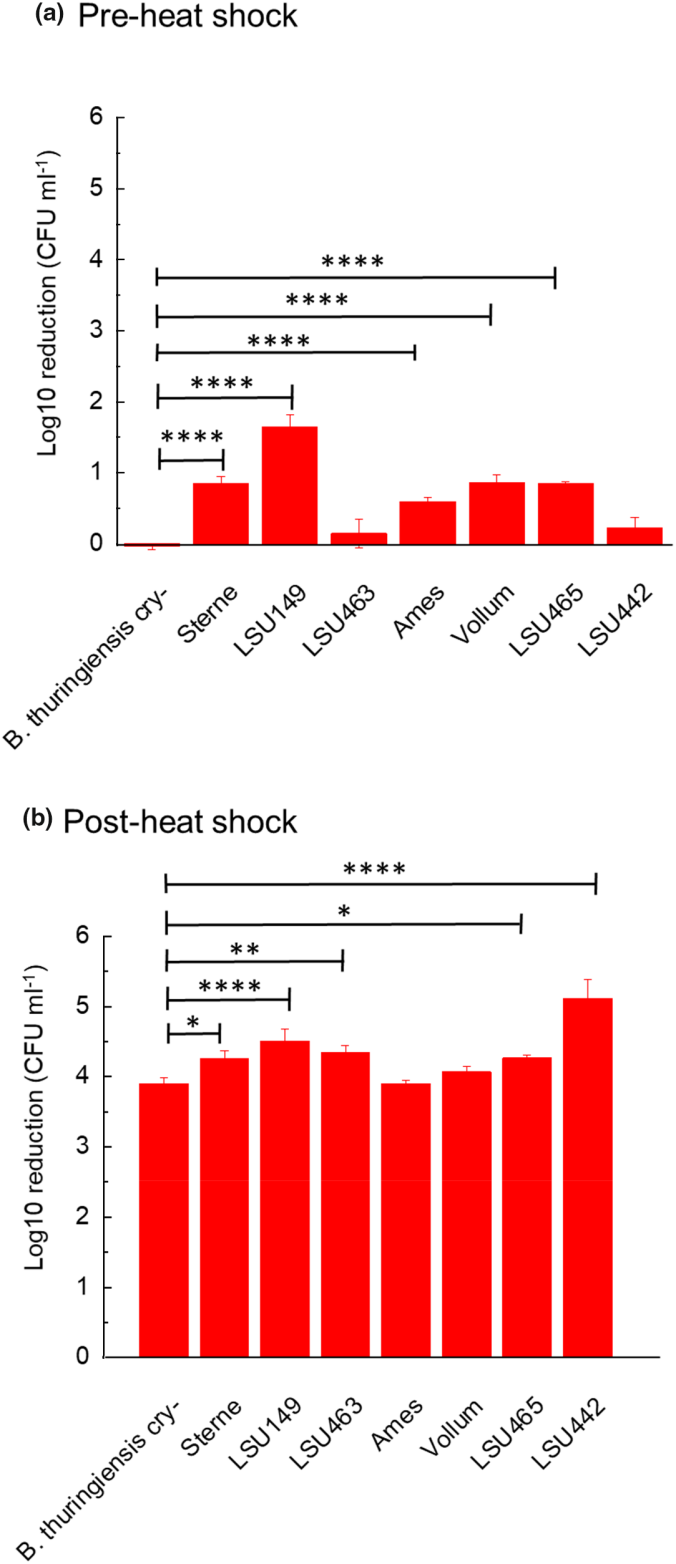
(a) Pre‐heat shock spore log10 reduction and (b) post‐heat shock spore log10 reduction of *Bacillus thuringiensis* cry‐ and seven *Bacillus anthracis* strains; Sterne; LSU149; LSU463; Ames, Vollum; LSU465 and LSU 442 following incubation for 24 h with phosphate buffered alanine and inosine germinant at 21°C (where *p ≤ 0.05, **p ≤ 0.01 and ****p ≤ 0.0001 using one‐way ANOVA with multiple comparison).

Further work compared germination efficacy of *B. thuringiensis cry‐* with seven *B. anthracis* strains, six of which represented the genetic diversity within *B. anthracis* determined by multiple‐locus VNTR (variable number tandem repeats) analysis (MVLA) sub‐clusters (Pilo & Frey, 2011). Figure 7a shows the pre‐heat shock total spore count for all strains when immersed in germinant. There was no significant decrease with *B. thuringiensis* cry‐ and LSU463 strains (compared with respective population controls at 0 h) over a 24 h incubation period. However, a reduction was observed for all other tested strains (*p* ≤ 0.05 for LSU442, *p* ≤ 0.0001 for Sterne, LSU149, Ames, Vollum and LSU465). The greatest reduction was seen in LSU149 (1.7 ± 0.2 log10 CFU per ml), followed by Vollum and LSU465 (0.9 ± 0.10 and 0.9 ± 0.03 log10 CFU per ml, respectively), Sterne (0.8 ± 0.1 log10 CFU per ml), and Ames (0.6 ± 0.1 log10 CFU per ml). Note the reduction in LSU442 was 0.2 ± 0.14 log10 CFU per ml, which may not be biologically significant.

Significantly higher germination rates were measured in every MVLA sub‐cluster *B. anthracis* strain compared with *B. thuringiensis* cry‐ following 24 h incubation at 21°C, with the exception of Ames and Vollum (Figure 7b). However, the average increase was only 0.4 log10 CFU per ml in three of these strains (Sterne, LSU463 and LSU465). The lowest level of germination was seen with *B. thuringiensis* cry‐ and Ames (both 3.9 log10 CFU per ml germination), the highest with LSU442 (5.1 log10 CFU per ml germination).

## DISCUSSION

The inoculation of surfaces with spores was carefully considered to produce a realistic challenge for the decontaminants and processes under investigation. Droplets of spore suspension drying on a surface can result in different spore deposition patterns depending on a range of factors that include droplet composition, size and shape (Richard et al., 2020), which can in turn affect decontaminant mass‐transport to the spore (solubility and diffusion) (Richard et al., 2020). Drying of larger droplets such as those that may be dispensed by pipetting can result in bacteria redistribution and aggregation to its peripheral ‘ring’ (Richard et al., 2020). Consequently, spores positioned internally within the aggregate may receive a lower dose of the active substance compared with relatively more dispersed spores from smaller droplets deposited by aerosol nebulization. In our work, representative porous and non‐porous materials found in the outdoor environment (steel, wood and cement) were inoculated with spores by aerosol nebulization because this is recognized as a realistic contamination mechanism (Klietmann & Ruoff, 2001).

It is important to note that while spores may be dispersed in suspension as single particles, this does not guarantee they will settle as a single layer of dispersed spores during surface nebulization. Images captured of spore inoculum nebulized onto glass slides confirmed largely deposition of single spore layers with areas of spore aggregation following nebulization onto glass (Figure 3c). However, factors including surface material properties, droplet‐wetting characteristics on the surface, nebulization time and environmental factors (temperature, humidity, airflow) can affect droplet size and droplet impact force and distribution on the surface. Therefore, understanding spore aggregation on the surface following the nebulization process is important and discussed in the following text.

To establish the nebulization parameters required to achieve a target spore density on a surface (initial spore concentration, volume for nebulization and nebulizer run‐time), we firstly nebulized a dye solution only onto steel surfaces and quantified the volume deposited per unit area by visible absorption spectroscopy. This measured volume was used to calculate the theoretical number of spores that would have been deposited for a defined initial inoculum. The method offered an accelerated approach to refining nebulization parameters, since the processing and analysis step (absorption spectroscopy) took a few minutes. In contrast, parameter optimization by direct nebulization of spores, would have required significantly more effort without any additional benefit.

The theoretical spore density calculated from the dye measurement was found to be 0.9 to 1.2 log10 CFU per cm^2^ lower than recoveries made from each surface type when nebulized with spores using the same operating parameters (Figure 3). This was likely because the theoretical spore density calculation assumed that the spores in suspension (for nebulization) were homogenously distributed. Consequently, the higher recoveries in the verification experiment were likely due to the effect of spore aggregation, despite the suspension being vortexed prior to use. Particle sizing of the spores during its production to ensure their sufficient separation, can be an important tool to optimize pellet resuspension by vortexing; particularly, since *B. thuringiensis* cry‐ has a hydrophobic exosporium (Setlow, 2014) affecting its solubility in water. Consequently, although the target spore inoculum was achieved, the dose–response data presented in this work were likely influenced to some extent by the potential shielding of the innermost spores of the aggregates from the formaldehyde.

The spore recoveries by surface type (Figure 3) demonstrated marginally lower recoveries from cement and wood compared with steel (mean −0.4 log10 CFU per cm^2^ at the 95% CI). Factors that may have contributed to this result, either in isolation or in combination were as follows: (a) differences in the affinity of surfaces to ‘capture’ spores as the aerosol flowed over the surface during nebulization; (b) porous surface offering higher surface area for spore adhesion and its resistance to mobilization during recovery process and (c) likewise, the relatively low roughness of the steel surface affording greater interaction between sand particles and spores for their mobilization into solution during the recovery process.

Dose–response methodology provides valuable information to optimize both practical considerations and efficacy in the event of a wide area biological contamination. As expected, our work with formaldehyde showed a general trend whereby higher decontaminant doses were needed to achieve a given level of hazard reduction as the surface became more porous (Figure 4a). However, a greater depth of information was achieved when the dose–response data were represented as three‐dimensional scatterplots (Figure 4b–d), or tabulated data calculated from the data points in Figure 4b–d (Table 3), which show the underpinning relationships between formaldehyde concentration, application volume and spore killing on each surface type for a fixed treatment time of 2 h.

A comparison of these plots showed that a mean low volume application of 1233 ± 58 L ha^−1^ (≡0.12 L m^−2^) onto steel surfaces using formaldehyde concentration of 30 g l^−1^, resulted in higher levels of spore killing (≥4 log10 CFU per cm^2^) compared with using a concentration of 10 g l^−1^ and application volumes of either higher or lower than mean 1233 ± 58 L ha^−1^ (*p* = 0.00 and *p* = 0.00, respectively, at 95% CI). In contrast, significantly larger volumes (mean 6623 ± 135 L ha^−1^ [≡0.66 L m^−2^]) at this high formaldehyde concentration (30 g l^−1^) was needed on cement surfaces to obtain the same level of reduction (*p* = 0.00). This is likely the result of a relatively lower contact time between the active‐substance and spores from a depleting effective surface concentration, when low volumes were applied to a porous surface such as cement. It was further supported by data obtained from the wood surface, which was relatively less porous compared with cement as determined by moisture retention readings using a protimeter (data not shown). Accordingly, Figure 4c and Table 3 demonstrate that a formaldehyde concentration of 30 g l^−1^ resulted in log10 reductions of ≥4 log10 CFU per cm^2^ using mean application volumes of ≥2600 ± 176 L ha^−1^, which were lower than the volumes required on cement (*p* = 0.00; mean difference 4023 L ha^−1^), but greater than those on steel (*p* = 0.00; mean difference 1367 L ha^−1^), to achieve the same level of spore killing (*p* = 0.53) with the same formaldehyde concentration. It is important to note that when inoculated surfaces were immersed into a volume of formaldehyde (30 g l^−1^) for the same treatment period (2 h), we obtained no detectable spore counts from all surfaces. This demonstrated that complete kill could be achieved in a 2 h contact time even on the cement surface if sufficient volume of a relatively low formaldehyde concentrate (30 g l^−1^) was applied to counter the absorptive losses.

The above findings are important considerations when decontaminating wide areas comprising different surface types and to ensure the efficient use of formulation, particularly if application is limited to a fixed concentration. However, surface porosity is not the sole factor influencing the effective dose at the surface of a material; further work would be required to verify the effect of surface roughness (effecting formulation wetting), organic content (material demand could reduce available formalin for spore interaction) or the effect of different meteorological conditions (temperature, humidity and solar irradiation) on the hazard reduction levels achieved employing the above application regimes. Such environmental and surface chemical demand factors have been studied under controlled test chamber conditions to determine their effect on the concentration of formalin when applied as a vapour for biological decontamination (Choi et al., 2021). In that study, there was no discolouration or change in physical appearance of material coupons (painted cinder block concrete and bare pine wood) or test chamber components upon formalin vapour exposure (Choi et al., 2021). The chemical compatibility of formalin is greater across a wide range of material types (polymers, metals, rubber) than common oxidant decontaminants, such as sodium hypochlorite (Cole Palmer Chemical Compatibility Database, 2022).

We used heat‐shock of vegetative cell/spore suspensions as a method to determine the proportion of spores that had successfully germinated following treatment with the germinant formulation. Therefore, an important control was to ensure spores were ‘hardy’ to the heat‐shock treatment so that a reduction in spore count following germinant exposure could be attributed to the effect of the germinant and not the intrinsic susceptibility of the spores to the heat‐shocking process. The data in Figure 4 confirmed that there was no significant effect of the heat‐shocking process on the viability of spores following their recovery from the surfaces, compared with non‐heat‐shocked spores (*p* = 0.98).

In our work, the germinant was buffered to neutral pH 7, partly due to surface material compatibility, but also because it was reported as optimum for L‐alanine induced germination of *B. cereus* (Broussolle et al., 2008; White et al., 1974) or inosine induced germination of this bacillus species (Broussolle et al., 2008). The effect of low pH on poor germination efficacy of *B. subtilis* was reported in the literature (Ciarciaglini et al., 2000).

The design of our experiments required inoculated surfaces to be immersed into an excess of germinant solution so that the surface was completely saturated during the 2 h incubation. This mitigated potential variability in the retention of l‐alanine and inosine at the surface (due to differences in surface porosity or evaporative losses) and the associated efficacy for inducing spore germination. Consequently, it ensured any gains or losses in formaldehyde dose–response could be readily resolved. Although this strategy met the needs of the study objectives, it must be noted that maintaining a surface saturated with germinant for 2 h under real world conditions is a logistically challenging requirement. Further work is needed to determine the level of germination achievable using more realistic spray applications.

Dose–response data in Figure 6 showed that higher levels of spore killing were achieved on inoculated steel for a given dose of formaldehyde when spores were pre‐germinated compared with ungerminated. Reducing the quantity of formaldehyde in the environment during a decontamination operation is essential to real‐world application due to its carcinogenic, toxic and corrosive properties, presenting both a contact and vapour hazard. The logistical burden associated with the additional volume used in the germination step could be offset by a more concentrated formaldehyde solution if non‐porous surfaces were to be treated but the strategy may not be applicable for surfaces of high porosity (such as the cement surfaces used in this study) for reasons discussed above. The latter needs to be further understood through dose–response experiments with sprayed germinant formulation. However, an alternative strategy to reduce volume of decontaminant may be to induce germination and allow for meteorological environmental factors conducive to desiccation, or solar irradiation, to inactive the germinated spores. However, if the formulation does not induce germination of the total spore population then careful consideration must be given to the potential spread of the ungerminated spore fraction in the open environment during this inactivation period.

If the efficacy of the germination approach was to be evaluated as a step in the decontamination strategy, it was important to understand that results achieved with the hazard group 1 test organism (*B. thuringiensis* cry‐) could be extrapolated with confidence to decontamination of virulent *B. anthracis* strains. The data in Figure 7a compared the pre‐heat shock total spore count for all virulent and non‐virulent strains following 24 h treatment with germinant. We found the *B. thuringiensis* cry‐ and LSU463 strains were less susceptible to killing during the germination process compared with all other strains for the tested conditions. These low levels of killing (0.6–1.7 log10 reduction) were likely a direct effect of germination induction without the nutrients available for microbial replication. Since both *B. thuringiensis* cry‐ and *B. anthracis* Ames strains showed the lowest germination of the strains tested, a germinant formulation and delivery strategy developed with these strains would likely provide greater efficacy when used against virulent strains of *B. anthracis*.

Although low levels of spore killing were measured in our work, alternative germinant formulations (based on l‐alanine and inosine) and suspension test conditions, used by other researchers reported relatively higher levels of inactivation. Buhr et al. (2019) demonstrated a 2.9 log10 reduction in *B. thuringiensis* cry‐ spores at 35°C, over 24 h, whereas no significant difference was observed using this strain in our study at 21°C over the same period and with an equivalent spore titre (7 log10 CFU per ml), Figure 7a. The formulation used by Buhr et al. included dipicolinic acid (40 mmol l^−1^), calcium chloride, buffered to pH 8 and with lower l‐alanine (100 mmol l^−1^) and inosine (1 mmol l^−1^) concentrations. One postulation may be that the survival of germinated *B. thuringiensis* cry‐ spores in the current study is related to incubation with 5× greater l‐alanine and 25× greater inosine at moderate temperature of 21°C compared with Buhr et al. (2019), which was germinated at 35°C.

This dose–response study represents the first of its kind to understand how concentration and volume of a sprayed decontaminant may affect spore killing on representative porous and non‐porous materials. Although these data provide operators with information to guide logistical planning if formaldehyde was used as the decontaminant, the data were collected at 21°C and did not evaluate the impact of other environmental factors that may be more conducive to higher evaporation potential. The initial benefits of germination to reduce the formaldehyde dose was demonstrated on a limited data set using inoculated steel. However, in these experiments germination was achieved under idealized conditions. Understanding the logistical impact of adding a pre‐germination step, where germinant solution must be applied by spray prior to decontaminant application, is needed to further assess the practical viability of the combined approach.

## CONFLICT OF INTEREST

No conflict of interest has been declared.
